# Modeling human glucose-6-phosphate dehydrogenase mutations using *C. elegans* GSPD-1

**DOI:** 10.17912/micropub.biology.000451

**Published:** 2021-09-10

**Authors:** Luiza N. Loges, Katherine M. Walstrom

**Affiliations:** 1 Division of Natural Sciences, New College of Florida, Sarasota, FL, USA; 2 Currently at Dept. of Global Health, Univ. of South Florida, Tampa, FL, USA

## Abstract

Glucose-6-phosphate dehydrogenase (G6PD) deficiency is an X-linked, recessive condition that causes intermittent jaundice or hemolytic anemia because of low NADPH levels in red blood cells. We performed steady-state enzyme kinetics with the recombinant *C. elegans* ortholog of human G6PD, GSPD-1, and two mutants containing amino acid changes found in human patients. The *K*_M_ values for glucose-6-phosphate were 100 ± 27 µM, 80 ± 22 µM, and 1000 ± 300 µM for the wild-type, D60N, and R252L GSPD-1 enzymes, respectively. The specific activities of the D60N and R252L mutants were 59% and 11%, respectively, of the wild-type value. Protein homology modeling suggested that the R252L mutation was more severe because the mutation caused a shift in the position of some active site residues. The D60N mutation may have affected the conformation of an outer loop of the enzyme. These data demonstrate that GSPD-1 is a promising model for human G6PD deficiencies, with the advantage that potential treatments could be studied *in vivo* in *C. elegans*.

**Figure 1. The D60N and R252L mutations in GSPD-1 reduce enzyme activity, and the R252L mutation appears to alter the position of the active site residues:**
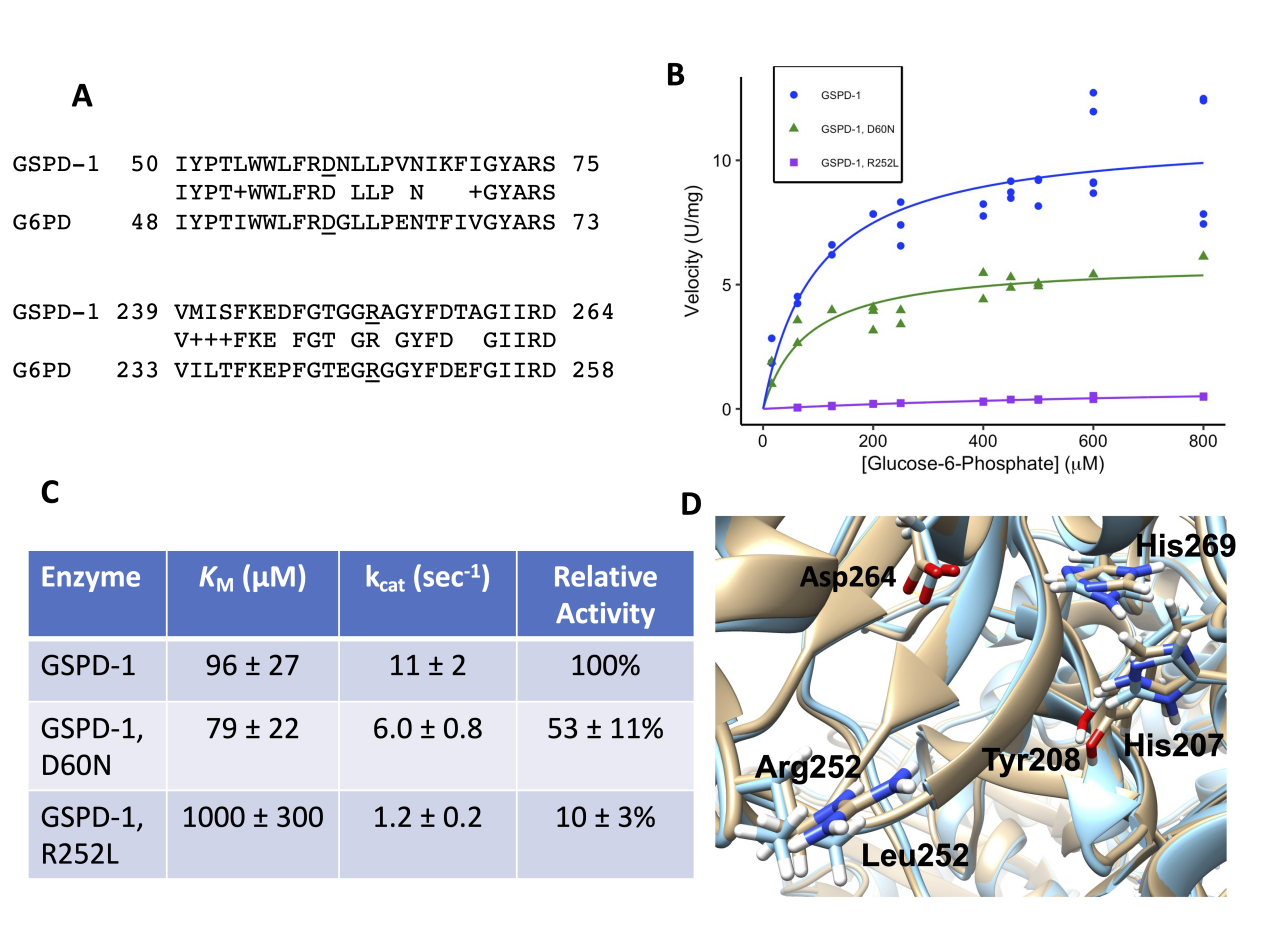
**(A)** The protein sequence alignments of *C. elegans* GSPD-1 (top line) and human G6PD (bottom line) surrounding the two mutation sites (underlined) are shown. The middle line of text shows identical amino acids, and a + symbol indicates a conservative substitution. The numbers show the amino acid positions at the ends of each sequence. **(B)** Michaelis-Menten graph of enzyme velocities (in μmol/min/mg or U/mg) for wild-type GSPD-1 (blue circles), the D60N mutant (green triangles), and the R252L mutant (purple squares) at varying glucose-6-phosphate (G6P) concentrations. All of the data points are shown, and the lines indicate the best-fit line. **(C)** This table shows the results from the Michaelis-Menten fits shown in B with standard errors. **(D)** View of the active site in GSPD-1 models. The wild-type structure is shown in beige, and the R252L structure is shown in light blue. Asp264 and His269 bind to the glucose moiety of G6P, His207 and Tyr208 bind to the phosphate moiety of G6P, and Arg252/Leu252 are at the site of the mutation. The positions of these amino acids in each structural model are shown.

## Description

Hundreds of different mutations in human glucose-6-phosphate dehydrogenase (G6PD) have been identified. Our goal was to test whether the *C. elegans* homolog, GSPD-1 (EC 1.1.1.49), was a good model for human G6PD mutants. The sequence of GSPD-1 (NP_502129) was aligned using BLASTP with human G6PD isoform b (NP_001035810.1) because this isoform encodes an active version of the protein (Altschul *et al.* 1990; Kanno *et al.* 1993). The sequences were 61% identical and 76% similar between amino acids 32-512 of G6PD and amino acids 34-520 of GSPD-1. We studied two mutations that in human G6PD were Asp58Asn in patients in Southern Italy and Arg246Leu in a patient in Tunisia (Vulliamy *et al.* 1988; Bendaoud *et al.* 2013). We chose these two mutants because they likely had residual enzyme activity, they had not been studied structurally before, and they were in residues that were conserved between the human and *C. elegans* orthologs. The protein sequence alignments between GSPD-1 and human G6PD in these two regions are shown in Figure 1A, and the corresponding mutations in GSPD-1 are D60N and R252L. The amino acids to the right of Asp60 are in an exterior loop, which may explain why these amino acids are less conserved than the surrounding amino acids, which are further inside the protein.

The recombinant, His-tagged versions of wild-type GSPD-1 and the D60N and R252L mutants were expressed in *E. coli* and purified. The protein concentrations of the wild-type, D60N, and R252L samples were 0.20 ± 0.017, 0.11 ± 0.019, and 0.50 ± 0.012 mg/mL, respectively, with the standard errors. Steady-state enzyme kinetics experiments were performed and analyzed using the Michaelis-Menten equation (Figures 1B and 1C). We found that the two mutant proteins had a lower specific activity than the wild-type enzyme, and the R252L mutant was more severely affected. We also found that the *K*_M_ for glucose-6-phosphate (G6P) for the D60N mutant (79 ± 22 μM) was similar to the wild-type value (96 ± 27 μM), while the *K*_M_ for the R252L mutant was increased (1000 ± 300 μM), suggesting that the binding of the substrate was affected in the R252L mutant.

In previous studies, the human G6PD D58N mutant had 12-39% of the wild-type activity (Vulliamy *et al.* 1988; Calabrò *et al.* 1990), compared to the GSPD-1 D60N mutant, which had 53 ± 11% of the wild-type activity (Figure 1C). The human G6PD R246L mutant had 25% of the wild-type activity (Bendaoud *et al.* 2013), compared to 10 ± 3% relative activity for the GSPD-1 R252L mutant. The enzyme kinetics method used for the human enzymes involved unpurified G6PD from lysed red blood cells diluted 1:20 into the reaction buffer (Betke *et al.* 1967). This differed from our method that used purified enzymes, although both reaction buffers were similar. Both methods found levels of G6PD activity for both mutants consistent with a class 3 or moderate to mild enzyme deficiency, defined as mutants having 10 – 60% of wild-type activity (Yoshida *et al.* 1971). The patient with the R246L mutation exhibited hemolytic anemia after ingesting fava beans, which is a common trigger of symptoms in G6PD-deficient patients, while the D58N patients were asymptomatic (Vulliamy *et al.* 1988; Bendaoud *et al.* 2013). The more severe symptoms caused by the R246L G6PD mutation are consistent with our finding that the R252L GSPD-1 mutant had very low activity.

To investigate how the protein structures might be affected by the mutations, we made protein structure homology models of wild-type GSPD-1 and the two mutants. We found that Asp60 is on an exterior loop of the enzyme. The protein homology model for wild-type GSPD-1 predicted a salt bridge between Asp60 and Arg440 in a neighboring exterior loop. The D60N model predicted a hydrogen bond (H-bond) between the side-chain of Asn60 and the backbone carbonyl group of Trp56. These results suggested that the D60N mutation eliminated the salt bridge with Arg440. The crystal structure of the human G6PD (PDB ID 6E07) had a salt bridge between Asp58 and Arg57 (which is also conserved in GSPD-1). The human enzyme had a Lys432 in the position corresponding to Arg440 in GSPD-1. Lys432 pointed toward Asp58 in human G6PD, but it was too far away to form an H-bond (5.54 angstroms). Therefore, this part of the wild-type GSPD-1 structure seemed to have a stronger salt bridge interaction between the two loops compared to the weaker ionic interaction in human G6PD. A superposition of the wild-type and D60N GSPD-1 models had nearly identical positions for the active site residues. This suggests that a change in the GSPD-1 structure caused by the loss of the Asp60-Arg440 salt bridge caused the modest effect of the D60N mutation on the GSPD-1 enzyme activity.

In GSPD-1, Arg252 lies on the outside of the protein near the active site (Figure 1D). The active site residues of two different G6PD orthologs were identified in three different crystal structures, with G6P bound in the active site of two of these structures (Cosgrove *et al.* 1998, 2000; Vu *et al.* 2021). These residues correspond to Asp264, His269, Asp206, and His207 in GSPD-1 based on sequence homology. Asp264 and His269 bind to the hydroxyl groups on C1, C2, and C3 of G6P, and both residues are near Arg252 (Cosgrove *et al.* 2000; Vu *et al.* 2021). In addition, His269 is likely the general base that removes the proton from the C1 hydroxyl group of G6P to initiate the G6PD reaction (Cosgrove *et al.* 1998). A comparison of the wild-type GSPD-1 and R252L homology models indicated that the R252L mutation caused a movement of the backbone, and this caused a ~0.8 angstrom shift in both Asp264 and His269 (Figure 1D). This could put the C1 of G6P out of a good alignment for the oxidation reaction. Movement of these active site residues could explain the decrease in enzyme activity and the increase in *K*_M_ that we observed for the R252L mutant. It seems unlikely that Arg252 binds to the phosphate group of G6P because the amino acids that bind to the phosphate (e.g., His207 and Tyr208 in Figure 1D) are on the other side of the active site (Cosgrove *et al.* 2000; Vu *et al.* 2021). Even though these phosphate-binding residues are not near Arg252 in the primary sequence, their positions were also shifted in the R252L model. The human G6PD structure has the same configuration around the active site, so GSPD-1 was a good model for this part of the human enzyme.

In summary, we found that protein homology models of *C. elegans* GSPD-1 had similar structures as human G6PD, and mutations associated with lower enzyme activity in human G6PD caused similar deficiencies in GSPD-1. Previous studies have shown that knocking down *gspd-1* expression by RNA interference (RNAi) caused a severe embryo hatching defect, and combining *gspd-1*(RNAi) with a mutation in cytosolic isocitrate dehydrogenase (*idh-1*) caused a growth and molting defect (Yang *et al.* 2013, 2019). These measurable phenotypes suggest that *in vivo* experiments and drug screenings could be performed with *C. elegans*, using either mutated GSPD-1 or a humanized version of this enzyme.

## Methods

The cDNA of GSPD-1 was amplified by RT-PCR from mRNA isolated from a mixed population of wild type Bristol N2 *C. elegans* (from the CGC) grown on OP50 *E. coli*. The PCR product and pET303/CT-His plasmid (Invitrogen) were cut with XbaI and XhoI and ligated with T4 DNA ligase (New England Biolabs, NEB). The plasmid added Leu-Glu to the C-terminus of GSPD-1 in addition to the six His residues. HiFi DNA assembly (NEB) was used to generate the mutant plasmids. All plasmid sequences were verified by dideoxynucleotide sequencing (Functional Biosciences, Inc.). The proteins were overexpressed in T7 Express *E. coli* (NEB) grown at 25 °C in Terrific Broth containing 100 μg/mL ampicillin. Protein induction was done with 0.01 mM IPTG for 15 hours at 25 °C. The cells were lysed on ice using a probe sonicator, and the proteins were purified using Bio-Rad Profinity^TM^ IMAC Ni-charged resin according to the manufacturer’s instructions. The protein purification was monitored by running samples on a 10% SDS-PAGE gel stained with Coomassie Blue, and the purified proteins had the expected molecular weights. The fractions with the most purified protein were dialyzed into 20 mM Tris-HCl, pH 8.0, 125 mM NaCl, 1 mM EDTA, 1 mM DTT, and 20% glycerol and stored at -20 °C. The protein samples had a small amount of high molecular-weight protein contaminants, but these contaminant bands were much lighter than the GSPD-1 bands. The protein concentrations were determined using a Coomassie Plus Bradford kit (Pierce™) with bovine serum albumin (Sigma) as the standard. The enzyme assays were done at 25 °C in 40 mM Tris-HCl, pH 8.0, 1 mM MgCl_2_, and 50 μM NADP^+^. The D-glucose-6-phosphate concentrations varied from 15 to 800 μM, and the rate of change of absorbance at 340 nm was measured to determine the rate of NADPH production. A higher concentration of mutant protein was added to the reactions so that the velocities were high enough to be precisely measured. Blank samples exhibited no activity. The enzyme kinetics data and Bradford data were analyzed and visualized using RStudio (version 1.4.1106).

The wild-type and mutant GSPD-1 protein sequences were submitted to SWISS-MODEL (Waterhouse *et al.* 2018), and the homology models were built using ProMod3, version 3.2.0 (Studer *et al.* 2021) with the crystal structure 6E07.1.A (Hwang *et al.* 2018) as a template. The models had GMQE and QMEAN scores (Studer *et al.* 2020) of 0.80 and -1.26 for wild-type GSPD-1, 0.80 and -1.29 for the D60N mutant, and 0.80 and -1.28 for the R252L mutant. These values indicated that the structures were good quality. The structures were refined by minimizing the free energy using the YASARA energy minimization server (Krieger *et al.* 2009). The z-scores for the minimized structures were calculated using the ProSA-web tools (Sippl 1993; Wiederstein and Sippl 2007). The resulting values (ranging from -9.95 to –9.68) were in the middle of the values for known protein crystal structures, suggesting that the models were good quality. The bond and rotamer angles of the minimized models were checked using MolProbity (Lovell *et al.* 2003; Williams *et al.* 2018). The models had 95-97% favored rotamers, 97-98% favored Ramachandran angles, and 3-6 Ramachandran outliers. None of these outliers were near the D60N or R252L mutation sites. The models also passed the Verify 3D assessment of protein models (Bowie *et al.* 1991; Lüthy *et al.* 1992; Eisenberg *et al.* 1997). The PyMOL Molecular Graphics system (Version 2.4.2, Schrödinger, LLC) was used to find the hydrogen bonds, and UCSF Chimera, version 1.15 (Pettersen *et al.* 2004), was used to make the active site figure.

## Reagents

The GSPD-1 wild-type and mutant plasmids and additional HiFi primer sequences used in this project are available upon request to walstrom@ncf.edu. The protein structure homology model PDB files are posted at Mendeley Data, V1, doi: 10.17632/mn3wf6v3wy.1 (https://data.mendeley.com/datasets/mn3wf6v3wy/1).

**Table:** 

**Strain**	**Genotype**	**Available from**
N2	*Caenorhabditis elegans*	CGC
T7 Express	*Escherichia coli*	New England Biolabs
		
**Plasmid**	**Genotype**	**Description**
pET303/CT-His		plasmid for expression of proteins with a C-terminal 6xHis-tag. Available from Invitrogen/ThermoFisher
		
**PCR primers**	**Sequence**	**Description**
*gspd-1* forward	CCGTGTCTAGA**ATG**GCATGCAAACGTCATTCAGTG	for *gspd-1* cDNA amplification, ATG is in bold and underlined.
*gspd-1* reverse	GTGGTGCTCGAG**TAG**CTTTGGAGCAACCCATTTGTATGT	for *gspd-1* cDNA amplification, final *gspd-1* Leu codon is in bold and underlined.
D60N mutagenic primer	CTGTGGTGGTTGTTCCGT**AAT**AACCTTTTGCCAGTCAAC	Primer used to introduce the D60N mutation in GSPD-1. Mutation site is in bold and underlined.
R252L mutagenic primer	GATTTTGGAACTGGTGGCC**TTG**CTGGATATTTTGATACA	Primer used to introduce the R252L mutation in GSPD-1. Mutation site is in bold and underlined.
		
